# Jaffe-Campanacci Syndrome: A Case Report and Review of the Literature

**DOI:** 10.7759/cureus.75726

**Published:** 2024-12-15

**Authors:** Furkan Erdoğan, Ercan Bayar, Bedirhan Albayrak, Mustafa Karal, Tolgahan Cengiz, İsmail Büyükceran, Nevzat Dabak

**Affiliations:** 1 Orthopedics and Traumatology, Ondokuz Mayis University, Samsun, TUR; 2 Orthopedics and Traumatology, Tosya State Hospital, Kastamonu, TUR; 3 Orthopedics and Traumatology, İnebolu State Hospital, Kastamonu, TUR

**Keywords:** cafe-au-lait spots, genetic syndromes, jaffe-campanacci syndrome, neurofibromatosis 1 (nf1), non-ossifying fibroma

## Abstract

Jaffe-Campanacci syndrome (JCS) is a rare disorder characterized by multiple non-ossifying fibromas (NOFs), café-au-lait spots, and other features such as mental retardation and cryptorchidism. It is often clinically and genetically similar to neurofibromatosis type 1 (NF1), complicating diagnosis. This report presents a 17-year-old male with right knee pain, café-au-lait spots, and axillary freckling. Radiographs revealed NOFs in the distal femur and proximal tibia. Surgery was performed, and pathological analysis confirmed NOFs, while genetic testing revealed a pathogenic NF1 mutation. JCS remains a poorly defined syndrome, with ongoing debate about its distinction from NF1. Surgical intervention is often necessary due to the high risk of pathological fractures in patients with large NOFs. This case supports the association between JCS and NF1 and highlights the importance of genetic testing in differential diagnosis. This case report also provides a brief literature discussion on the very rare syndrome JCS.

## Introduction

Jaffe-Campanacci syndrome (JCS) was first described by Jaffe [1] in 1958 and Campanacci [2] and later by Mirra et al. in 1982 [3], as a series of symptoms and signs characterized by multiple non-ossifying fibromas (NOFs), café-au-lait spots, and giant cell granulomas of the mandible. Additional symptoms associated with this syndrome include mental retardation, hypogonadism, cryptorchidism, various congenital eye anomalies, and cardiovascular malformations [2]. In cases of JCS, NOFs are predominantly located in the metaphyseal region and tend to be larger than those seen in isolated NOF cases. Multiple anatomical areas are affected, and cortical irregularities with minor fractures may coexist [4]. The most commonly involved areas are the distal femur, proximal and distal tibia, and proximal humerus, where significant bone deformities may occur due to the size of the lesions [4].

JCS is a syndrome that shares clinical similarities with neurofibromatosis type 1 (NF1), and its distinction in the literature remains unclear. The clinical diagnosis of NF1 is typically based on the presence of multiple café-au-lait spots, freckling in the axillary or inguinal regions, multiple neurofibromas, Lisch nodules, and confirmed NF1 gene mutations in first-degree relatives [5]. Given these reports, differentiating JCS and NF1 based on symptoms and phenotypic characteristics is often challenging. Case reports in the literature suggest that JCS and NF1 may not be distinguishable clinically, genetically, or phenotypically [5]. In this rare case report, we will examine a young patient diagnosed with the rare disease JCS who had NOF detected as a result of right knee pain and accompanying symptoms such as cafe-au-lait spots, axillary freckling, and mental retardation.

## Case presentation

**Figure 1:**
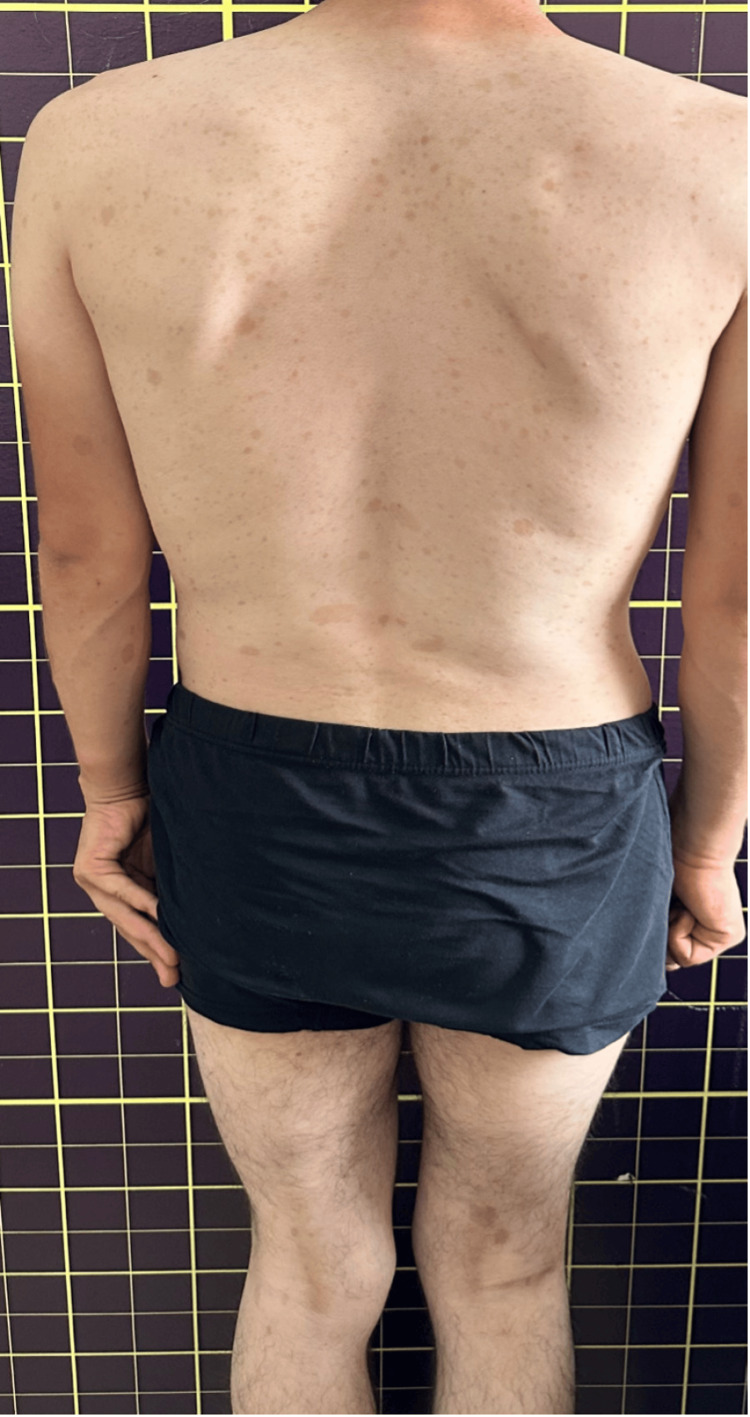
Diffuse cafe-au-lait spots on the patient's body

**Figure 2:**
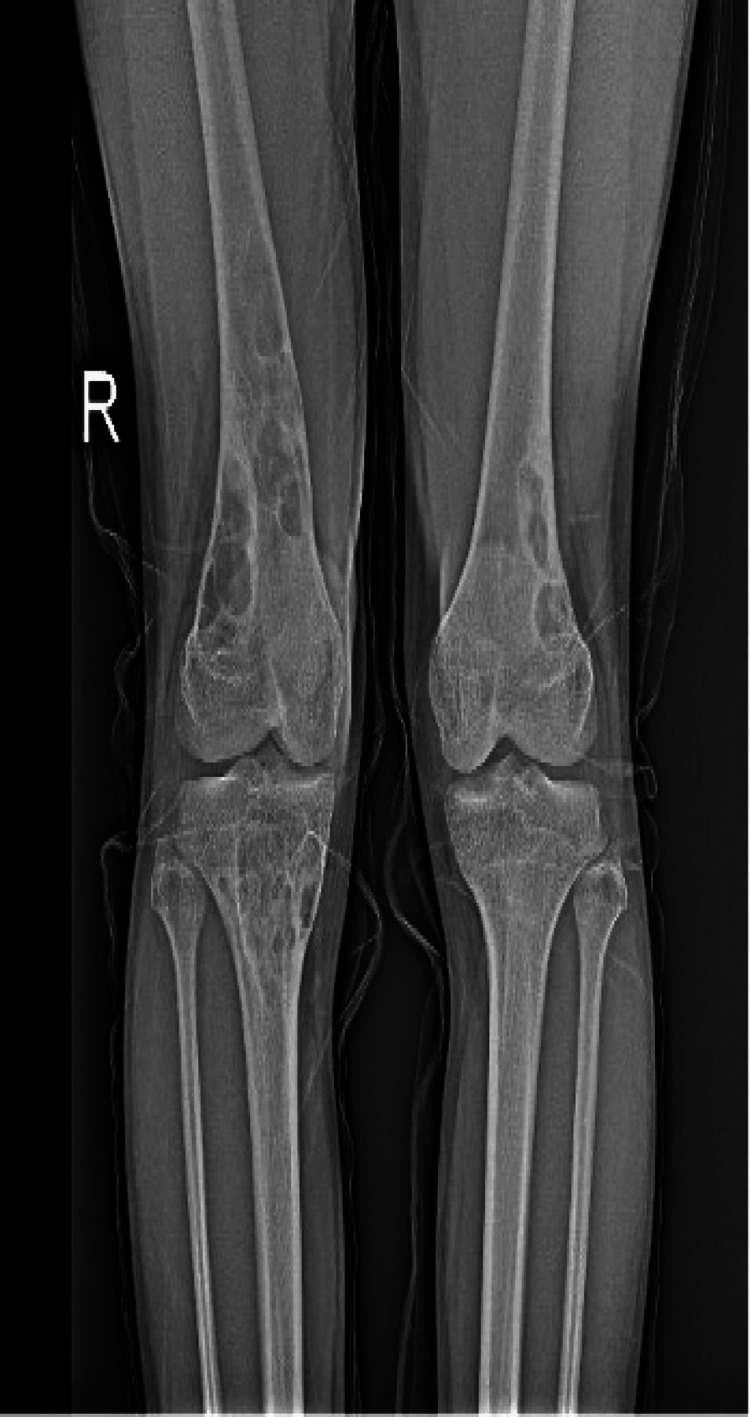
Radiographs showed multiple NOFs NOFs: non-ossifying fibromas

**Figure 3:**
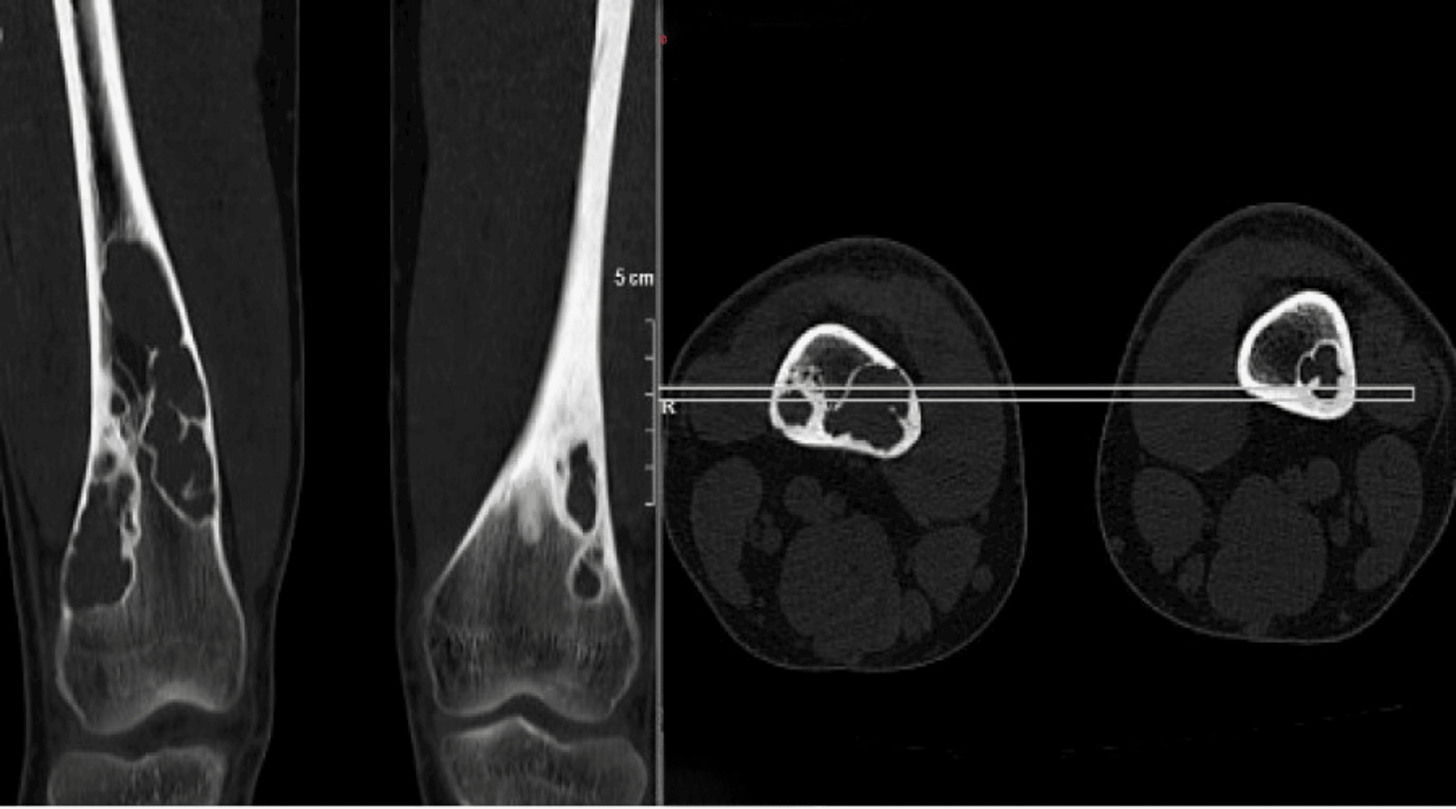
CT images of NOF in both femurs of the patient CT: computed tomography, NOF: non-ossifying fibroma

A 17-year-old male patient presented to the orthopedic and traumatology clinic with pain in the distal right femur. Physical examination revealed multiple café-au-lait spots and axillary freckling on the skin (Figure 1). The patient was 170 cm tall and weighed 57 kg. He had a slightly broad nose with thickened nasal wings. His medical history included mild intellectual disability. No abnormalities were observed in laboratory findings. No dermal lesions suggest neurofibromas or schwannomas, and no Lisch nodules were detected in the eyes. Bilateral femur and right tibia radiographs showed multiple NOFs (Figures 2-3). No lesions were found in the other extremities.

Surgery was planned to address the pain in the distal right femur. The surgical procedure included curettage, grafting, and osteosynthesis with a plate and screws. A 10 cm incision was made laterally on the right femur to reach the lesion. A cortical bone flap was opened at the lesion site, and the lesion was curetted and sent for pathological examination. After grafting from the iliac crest and replacing the cortical flap, osteosynthesis with a plate and screws was performed. Genetic analysis was requested from the excised material. The genetic test revealed a pathogenic heterozygous mutation in the NF1 gene, precisely a c.3758_3762del DNA sequence.

**Figure 4:**
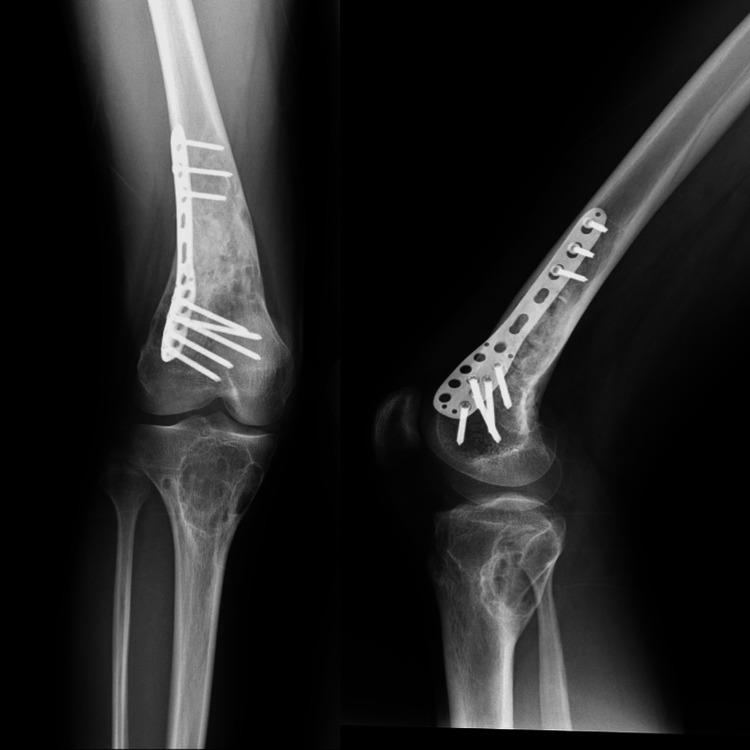
Postoperative radiograph of the patient taken at six months

Following the surgical procedure, the patient was immobilized in a splint for one month, after which partial weight-bearing mobilization was initiated. Total weight-bearing mobilization was started two weeks later, and the patient reported no pain during this period. After two months, the patient could mobilize without pain and had no discomfort in other extremities. The pathological examination showed a storiform pattern with multinucleated giant cells and spindle cells surrounding areas of bleeding, along with histiocytes loaded with hemosiderin. These findings were consistent with a diagnosis of NOF. The genetic test confirmed a pathogenic heterozygous mutation in the NF1 gene, c.3758_3762del DNA sequence. The patient's postoperative follow-up continues, and the sixth-month radiographic image is given in Figure 4.

## Discussion

Today, according to most scientists, JCS is considered a poorly defined syndrome associated with multiple NOFs, skin findings (café-au-lait macules), and non-skeletal anomalies such as mental retardation, hypogonadism, cryptorchidism, ocular abnormalities, or cardiovascular malformations. Whether JCS is a specific form of NF1 or a separate syndrome is still debated. When Jaffe first described this syndrome, he suggested that it was a different subtype of NF1 [1]. Mirra et al. also reported that JCS was a distinct form of NF1 [3]. However, studies conducted over the years suggested that JCS and NF1 are different disease entities [6,7]. In 2013, the World Health Organization classified JCS under bone and soft tissue disorders as NOFs associated with NF1 [8]. Other studies reported multiple NOFs, a distinguishing feature of JCS, in NF1 patients [9,10]. In a study by Stewart et al., a pathogenic NF1 mutation was found in most patients with café-au-lait macules and NOFs, suggesting that many JCS cases may indeed be associated with NF1 [11]. In the case we present, genetic analysis of the NOFs revealed a heterozygous pathogenic mutation in the NF1 gene, supporting the hypothesis that JCS cases are related to NF1 syndrome.

Clinically, JCS often presents with skin lesions such as café-au-lait macules or axillary freckling, along with multiple NOFs. Other features may include cardiovascular malformations, intellectual disability, cryptorchidism, and hypogonadism [3,6]. Our patient exhibited multiple café-au-lait macules and axillary freckling, in addition to multiple NOF lesions and mild intellectual disability. The patient also had a ptotic phenotype, with broad and thickened nasal wings. Some authors suggest that axillary freckling is a significant finding for JCS [12]. Colby and Saul published a case series of four patients with facial photographs, noting a mildly ptotic phenotype, a slightly broad nose, and thickened nasal wings [9]. The literature on patients with JCS supports our case's clinical and phenotypic features.

JCS affects multiple anatomical regions through its characteristic multiple NOF lesions. The most common locations for NOFs are the distal femur, proximal tibia, and, less frequently, the proximal humerus, fibula, and radius [4,9,13]. In our patient, multiple NOF lesions were present in the distal femur and proximal tibia. Our findings align with the literature, and NOFs' characteristics are significant. In JCS cases, NOFs are typically located in the metaphysis, tend to be significant, and have been reported to create fibrous defects in the cortex [4]. The characteristics of the NOFs in our case are consistent with the literature. Diagnosing NOFs pathologically in JCS is essential to prevent diagnostic errors. Histologically, NOFs contain variable numbers of multinucleated giant cells, hemosiderin-laden histiocytes, and spindle-shaped fibroblasts in a storiform fibrous tissue pattern [14]. The pathological diagnosis in our patient was consistent with these findings.

There is no consensus on the treatment of JCS. Most cases are treated conservatively. However, if there is a risk of pathological fracture, if the lesion is painful, or if progressive deformity is expected, surgical treatment should be considered. Surgical options include curettage, grafting, and internal fixation of NOFs [9]. Amputation has been reported in a case with severe deformity [15]. In 59.1% of 22 JCS cases reported in the literature, pathological fractures were present, a much higher incidence than pathological fractures in isolated NOF cases [15]. Therefore, surgical intervention is often required in JCS cases. Another study reported that radiographic diagnosis of NOFs can be challenging. Thus, a two-stage surgical procedure is recommended, with an open biopsy first followed by definitive procedures after confirmation of the diagnosis [16]. In our patient, pain in the right femur was present, and due to the risk of pathological fracture, curettage, grafting, and internal fixation were performed. No complications were encountered during the patient's one-year follow-up.

## Conclusions

JCS is a poorly defined syndrome associated with multiple NOFs, skin manifestations (café-au-lait spots and axillary freckling), and some features of NF1. NOF lesions are typically large and frequently located in the distal femur and proximal tibia. Genetic analysis of the NF1 gene using biopsy material from NOF lesions is recommended. While conservative approaches can be accepted in treatment, the high incidence of pathological fractures warrants regular assessment of this risk, and surgical options should also be considered. During internal fixation, curettage and bone grafting should be considered to promote healing.

## References

[1] Jaffe HL (1959). Tumors and tumorous conditions of the bones and joints. Acad Med.

[2] Campanacci M, Laus M, Boriani S (1983). Multiple non-ossifying fibromata with extraskeletal anomalies: a new syndrome?. J Bone Joint Surg Br.

[3] Mirra JM, Gold RH, Rand F (1982). Disseminated nonossifying fibromas in association with café-au-lait spots (Jaffe-Campanacci syndrome). Clin Orthop Relat Res.

[4] Mankin HJ, Trahan CA, Fondren G, Mankin CJ (2009). Non-ossifying fibroma, fibrous cortical defect and Jaffe-Campanacci syndrome: a biologic and clinical review. Chir Organi Mov.

[5] Stumph DA (1988). Neurofibromatosis. Conference statement, National Institute of Health development conference. Arch Neurol.

[6] Steinmetz JC, Pilon VA, Lee JK (1988). Jaffe-Campanacci syndrome. Journal of Pediatric Orthopaedics.

[7] Al-Rikabi AC, Ramaswamy JC, Bhat VV (2005). Jaffe-Campanacci syndrome. Saudi medical journal.

[8] World Health Organization (2013). WHO classification of tumours of soft tissue and bone. WHO classification of tumors of soft tissue and bone: WHO classification of tumours, vol. 5.

[9] Colby RS, Saul RA (2003). Is Jaffe-Campanacci syndrome just a manifestation of neurofibromatosis type 1?. Am J Med Genet A.

[10] Schotland HM, Eldridge R, Sommer SS, Malawar M (1992). Neurofibromatosis 1 and osseous fibrous dysplasia in a family. Am J Med Genet.

[11] Stewart DR, Brems H, Gomes AG, Ruppert SL, Callens T, Williams J (2014). Jaffe-Campanacci syndrome, revisited: detailed clinical and molecular analyses determine whether patients have neurofibromatosis type 1, coincidental manifestations, or a distinct disorder. Genet Med.

[12] Cherix S, Bildé Y, Becce F, Letovanec I, Rüdiger HA (2014). Multiple non-ossifying fibromas as a cause of pathological femoral fracture in Jaffe-Campanacci syndrome. BMC musculoskeletal disorders.

[13] Fauré C (1986). Multiple and large non-ossifying fibromas in children with neourofibromatosis. Pediatr Radiol.

[14] Schajowicz F, Schajowicz F (1993). Histological classification of bone tumours. Histological typing of bone tumours.

[15] Jiang J, Liu M (2024). Jaffe-Campanacci syndrome resulted in amputation: a case report. World J Clin Cases.

[16] Sonar M, Isik M, Ekmekci AY, Solmaz OA (2012). Pathological fractures on both lower limbs with Jaffe-Campanacci's syndrome. BMJ Case Rep.

